# Biofilm formation and antibiotic sensitivity in *Elizabethkingia anophelis*

**DOI:** 10.3389/fcimb.2022.953780

**Published:** 2022-07-28

**Authors:** Shaohua Hu, Yan Lv, Hao Xu, Beiwen Zheng, Yonghong Xiao

**Affiliations:** ^1^State Key Laboratory for Diagnosis and Treatment of Infectious Diseases, National Clinical Research Center for Infectious Diseases, Collaborative Innovation Center for Diagnosis and Treatment of Infectious Diseases, The First Affiliated Hospital, Zhejiang University School of Medicine, Hangzhou, China; ^2^Department of Blood Transfusion, The First Affiliated Hospital, Zhejiang University School of Medicine, Hangzhou, China; ^3^Department of Structure and Morphology, Jinan Microecological Biomedicine Shandong Laboratory, Jinan, China

**Keywords:** *Elizabethkingia anophelis*, nosocomial infections, multidrug-resistant, biofilm formation, biofilm-specific resistance

## Abstract

*Elizabethkingia anophelis* has recently gained global attention and is emerging as a cause of life-threatening nosocomial infections. The present study aimed to investigate the association between antimicrobial resistance and the ability to form biofilm among *E. anophelis* isolated from hospitalized patients in China. Over 10 years, a total of 197 non-duplicate *E. anophelis* strains were collected. Antibiotic susceptibility was determined by the standard agar dilution method as a reference assay according to the Clinical and Laboratory Standards Institute. The biofilm formation ability was assessed using a culture microtiter plate method, which was determined using a crystal violet assay. Culture plate results were cross-checked by scanning electron microscopy imaging analysis. Among the 197 isolates, all were multidrug-resistant, and 20 were extensively drug-resistant. Clinical *E. anophelis* showed high resistance to current antibiotics, and 99% of the isolates were resistant to at least seven antibiotics. The resistance rate for aztreonam, ceftazidime, imipenem, meropenem, trimethoprim-sulfamethoxazole, cefepime, and tetracycline was high as 100%, 99%, 99%, 99%, 99%, 95%, and 90%, respectively. However, the isolates exhibited the highest susceptibility to minocycline (100%), doxycycline (96%), and rifampin (94%). The biofilm formation results revealed that all strains could form biofilm. Among them, the proportions of strong, medium, and weak biofilm-forming strains were 41%, 42%, and 17%, respectively. Furthermore, the strains forming strong or moderate biofilm presented a statistically significant higher resistance than the weak formers (p < 0.05), especially for piperacillin, piperacillin-tazobactam, cefepime, amikacin, and ciprofloxacin. Although *E. anophelis* was notoriously resistant to large antibiotics, minocycline, doxycycline, and rifampin showed potent activity against this pathogen. The data in the present report revealed a positive association between biofilm formation and antibiotic resistance, which will provide a foundation for improved therapeutic strategies against *E. anophelis* infections in the future.

## Introduction

*E. anophelis* is an emerging pathogen that can pose a significant threat to patients due to its unclear mechanism of antibiotic resistance and high mortality rate among nosocomial isolates (Janda and Lopez, 2017; Lin et al., 2019). *E. anophelis* is a class of Gram-negative, non-fermenting bacillus that is ubiquitously recovered from hospital environments (Kyritsi et al., 2018; Nicholson et al., 2018; Choi et al., 2019; Lee et al., 2021). Unexpectedly, it has been reported that the bacterium can be isolated from contaminated corona virus disease 2019 (COVID-19) swab kits (Xu et al., 2022). Moreover, it is related to mainly immunocompromised patients and has been clinically identified as one of the most important opportunistic pathogens responsible for nosocomial infections or healthcare-associated infections (Janda and Lopez, 2017; Lin et al., 2019). Since the first *E. anophelis* meningitis case was reported in 2012 (Frank et al., 2013), an increasing number of infections have been reported recently, including bacteremia, pneumonia, meningitis, catheter-related bloodstream infections, skin and soft-tissue infections, urinary tract infections, and eye infections (Lau et al., 2016; Hu et al., 2017; Bulagonda et al., 2018; Chew et al., 2018; Nielsen et al., 2018; Lin et al., 2018a; Auffret et al., 2021). In addition, several life-threatening outbreaks of infections caused by *E. anophelis* have successively been described in many regions worldwide, including Singapore, the United States, Hong Kong, Taiwan, and South Korea (Teo et al., 2013; Navon et al., 2016; Perrin et al., 2017; Lin et al., 2018a; Choi et al., 2019). In addition, in previous studies, it has been revealed that the incidence of *E. anophelis* infection was highly underestimated due to its frequent misidentification as *Elizabethkingia meningoseptica* by conventional laboratory identification methods (Lau et al., 2016; Lin et al., 2017; Kelly et al., 2019). Undoubtedly, such a high underestimation and mortality rate of *E. anophelis* infections cause a tremendous burden on a country’s health system.

It has been documented that *E. anophelis* is notorious for its high resistance to many antibacterial drugs, including penicillins, cephalosporins, carbapenems, aminoglycosides, tetracyclines, and β-lactamase inhibitors (Lin et al., 2018a; Lin et al., 2018b; Wang et al., 2019; Chiu et al., 2021; Larkin et al., 2021; Tang et al., 2021). Several investigations have revealed that *E. anophelis* isolates usually express resistance to multiple current commonly used antibiotics. In contrast, results from other studies have indicated susceptibility to several antibacterial agents, such as certain β-lactams, carbapenems, aminoglycosides, fluoroquinolones, or sulfa antibiotics (Teo et al., 2013; Navon et al., 2016; Han et al., 2017; Perrin et al., 2017; Lin et al., 2018a; Choi et al., 2019; Wang et al., 2020; Chiu et al., 2021). These inconsistent antimicrobial susceptibility testing (AST) patterns may be attributed to an insufficient sample size and the various origins of the strains in different countries and regions. Limited drug susceptibility test data are available for this bacterium, especially in mainland China. Therefore, further thorough exploration of drug resistance in *E. anophelis* from diverse sources is of utmost importance.

Biofilm is defined as the microbial population consisting of groups of bacterial cells, which are adherent to a surface and are comprised within a self-produced extracellular matrix, including proteins, extracellular DNA, and polysaccharides (Harika et al., 2020; Hashemzadeh et al., 2021). Bacterial cells within the biofilm are highly coordinated and undergo phenotypic switches to generate communities that are resistant to an adverse external environment (Shenkutie et al., 2020). Such a phenotype switch can also contribute to the emergence of antibiotic resistance by encoding antibiotic resistance genes, genetic mutation, restricting antibiotics, or counteracting host immunity (Shenkutie et al., 2020). Nearly all multidrug-resistant (MDR) Gram-negative bacteria and their virulence factors are persistent problematic responses in hospitalized patients during biofilm production (Husain et al., 2021). Among them, the indwelling device is the most important in biofilm formation and colonization (Karami et al., 2020). Biofilms protect bacteria from the host immune system and antimicrobial agents. For example, the formation of biofilms by *P. mirabilis* strengthens the complexity of bacterial resistance, prolongs treatment time, and further intensifies the infection ability (Ranjbar-Omid et al., 2015). The bacteria became very robust against all available bacteriostatic agents, and the underlying mechanisms involved were developed. Hence, it is important to create connections between biofilm production and drug resistance in clinical isolates of *E. anophelis*.

In recent years, the formation of biofilm by *Elizabethkingia* species has been discussed in a few studies (Jacobs and Chenia, 2011; Tang et al., 2021). However, too little research data are available, and no study has reported a correlation between biofilm formation and antibiotic resistance in *Elizabethkingia* species from humans. Therefore, the association between the biofilm formation capability and antibiotic resistance in clinical *E. anophelis* isolates is unknown. Here, we used the Clinical and Laboratory Standards Institute (CLSI)–recommended standard agar dilution method as a reference assay and examined the antimicrobial susceptibility results for 197 clinical *E. anophelis* isolates to fill those research gaps. The present study aimed to study biofilm formation and different antibiotic sensitivity in *E. anophelis* strains that cause nosocomial infections in China and present any possible link between the ability to form biofilm and MDR. To the best of our knowledge, this is the first study that investigated biofilm formation and a correlation between antibiotic resistance in *E. anophelis* isolates. Data on the phenotypic characterization of the biofilm-forming capacity and the correlation between antibiotic susceptibility may offer valuable insights into the development of medication and preventive strategies for *E. anophelis* nosocomial infections.

## Materials and methods

### Sampling and bacterial isolation

The database of the Clinical Strain Library of the First Affiliated Hospital of Zhejiang University School of Medicine was searched from January 2010 to April 2019 for microbial cultures that yielded *E. anophelis*. The collected isolates were kept in brain-heart infusion broth (Oxoid, UK) containing 20% glycerol at −80°C until use. All 197 isolates used in this study were routinely collected from patients according to their clinical requirements. All used strains of *E. anophelis* species that were previously collected from blood, sputum, abdominal fluid, cerebrospinal fluid (CSF), bronchoalveolar fluid (BAL), urine, other soft tissue, etc. Samples were mainly collected from patients in the intensive care unit, hematology department, infectious disease department, and surgical ward. Of these patients, 76.4% (149 of 195) were men and 23.6% (46 of 195) were women. Detailed data for two isolates were unavailable. The mean age of the patients was 61 ± 18 years. Among them, except for two babies, none of the patients were under 18 years of age, and 76 patients were over 50 years of age. Isolates were incubated at 37°C for 24 h from the −80°C stock in Mueller–Hinton Agar (MHA) (Oxoid, UK) without antibiotics in aerophilic conditions. Microbial isolates derived from patients were initially identified using conventional tests by a matrix-assisted laser desorption ionization time-of-flight mass spectrometry (Bruker Daltonics, USA) and were verified by genomic average nucleotide identity analysis.

### Antimicrobial susceptibility testing

Agar dilution techniques determined antimicrobial sensitivity testing according to the procedures described in the CLSI guidelines (2020). The antimicrobial resistance of the isolates to 19 antibiotics (Meilunbio, China), including piperacillin, piperacillin-tazobactam, ceftazidime, cefepime, imipenem, meropenem, aztreonam, gentamicin, amikacin, minocycline, doxycycline, tetracycline, tigecycline, ciprofloxacin, levofloxacin, trimethoprim-sulfamethoxazole, rifampin, vancomycin, and chloramphenicol, was investigated. Susceptible, intermediate, and resistant interpretation was based on the CLSI guidelines for “other non-Enterobacteriaceae”. The susceptibility criteria for tigecycline were interpreted according to “Enterobacteriaceae breakpoints” (susceptible, ≤ 2 mg/L; intermediate, = 4 mg/L; resistant, ≥ 8 mg/L), provided by the US Food and Drug Administration (Chiu et al., 2021). Moreover, for rifampin and vancomycin, the susceptibility testing results and minimum inhibitory concentration (MICs) were interpreted according to the “*Enterococcus* species” of the CLSI standards for rifampin and vancomycin. Bacteria *E. coli* ATCC 25922, *Pseudomonas aeruginosa* ATCC 27853, and *Staphylococcus aureus* ATCC 29213 were used as quality control strains. The standardized definition of MDR, extensively drug-resistant (XDR), and pan-drug-resistant (PDR) bacteria has been well studied. MDR strains were defined as strains that acquired non-susceptibility to at least one agent per three or more antimicrobial categories, XDR strains were defined as non-susceptible to at least one agent in all but two or fewer antimicrobial classes, and PDR strains were defined as non-susceptible to all agents in all antimicrobial categories (Magiorakos et al., 2012).

### Biofilm formation and quantification assay

Biofilm-forming capacities of the isolates were evaluated in triplicate using the crystal violet method for *Elizabethkingia* species as previously described with modifications (Jacobs and Chenia, 2011; Tang et al., 2021). Briefly, overnight grown *E. anopheli*s [cultured at 37°C in 2 ml of Mueller–Hinton Broth (MHB) (Oxoid, UK)] was harvested. Then, the cultures were diluted in MHB medium adjusted approximately to 0.5 McFarland. A 20-μl aliquot of each suspension was then diluted 1:10 in 180 μl of MHB in a 96-well cell culture-treated polystyrene plate (Corning Incorporated, USA). Following 24 h of growth at 37°C overnight, plates were washed three times with 200 μl of phosphate-buffered saline (PBS; 0.01mol/L) to remove unattached bacteria. After air drying, adherent cells were fixed with 200 μl of methanol for 15 min and stained with 200 μl of 1% crystal violet (Beyotime, China) for 15 min at room temperature. The staining solution was removed, and the plate was washed three times with 200 μl of PBS (0.01mol/L). After removing the washing solution, 150 μl of 33% acetic acid was added to each well to dissolve the biofilm-bound crystal violet and incubated for 5 min on a shaking table. The optical density (OD) of each well was measured at 595 nm using a microtiter plate reader Epoch2 (BioTek, USA). The OD at 595 nm was obtained as an index of adherent bacteria and biofilm formation. The OD value of sterile medium with fixative and dye was recorded and subtracted from the results to determine the background OD. All strains were classified into the following four categories: the first category comprised those not considered biofilm producers when OD595 ≤ ODc (the mean OD of the negative control). The other three were weak biofilm formation, OD595 >ODc-2XODc; moderate biofilm formation, OD595 >2XODc–4XODc; and strong biofilm formation, OD595 >4XODc.

### Scanning electron microscopy analysis

Scanning electron microscopy imaging (SEM) analysis was performed in the State Key Laboratory of Rice Biology of Zhejiang University, using a Scanning Electron Microscope (TM 4000 PLUS, HITACHI, Tokyo, Japan). Bacteria were incubated for 24 h at 37°C with 15 ml of MHB under shaking conditions. After centrifugation, the precipitated bacteria were immediately fixed in 2.5% fresh glutaraldehyde and fixed for 2 h. Next, bacteria were rinsed three times with distilled water (centrifugal discard supernatant at each step, distilled water was added, and clots were blown with a straw). Then, dehydration was performed with increasing concentration of ethanol: 20 min at 50%, 20 min at 75%, 20 min at 85%, 20 min at 95%, and two times for 20 min in 100% ethanol prior to crucial point drying. Subsequently, critical point drying, ion sputtering, and microscope observation were carried out successively.

### Statistical analysis

Statistical analyses were performed using SPSS version 23.0 software (IBM, Armonk, NY, USA). To examine the effect of biofilm production on the susceptibility of the strains, data normality of continuous variables was initially verified using the Shapiro–Wilk test. The t-test and Mann–Whitney U test were used to compare the differences between the two groups. The Kruskal–Wallis test was employed for multiple comparisons. The statistical significance was set at P < 0.05.

## Results

### Antimicrobial susceptibility patterns

The susceptibility to 19 antimicrobials of human clinical significance was investigated in all 197 clinical *E. anophelis* isolates. According to the CLSI-recommended agar dilution method, drug susceptibilities of the *E. anophelis* isolates and MIC ranges for all 19 tested antimicrobials are presented in **Table 1** and **Table S1**. MDR was observed in all 197 *E. anophelis* isolates (**Table 1**). Ten percent of pan-resistant strains (XDR) was detected among them. All strains presented intermediate susceptibility to six antibiotics and presented no susceptibility to at least four antibiotics. Of the tested antibiotics, *E. anophelis* isolates showed low varying degrees of MDR. However, none of the strains of *E. anophelis* investigated was pan-susceptible (susceptible to all antimicrobials tested), whereas two isolates (1% of the isolates; one strain from sputum and one from fluid) were resistant to only four antibiotics.

**Table 1:** Antimicrobial MICs (mg/L) and susceptible rates of 197 *E. anophelis* isolates determined using the agar dilution.

Antimicrobial agent	Susceptibility testing assay		
	Agar dilution (mg/L)	MIC range	S no. (%)	I no. (%)	R no. (%)
Piperacillin	0.5–256	8–>256	49 (24.87)	50 (25.38)	98 (49.75)
Piperacillin-tazobactam	0.5–256+4	4/4–>256/4	67 (34.01)	73 (37.06)	57 (28.93)
Ceftazidime	0.25–64	4–>64	1 (0.51)	0 (0)	196 (99.49)
Cefepime	0.06–64	2–>64	2 (1.02)	7 (3.55)	188 (95.43)
Imipenem	0.03–32	0.25–>32	1 (0.51)	0 (0)	196 (99.49)
Meropenem	0.008–32	0.06–>32	2 (1.02)	0 (0)	195 (98.98)
Aztreonam	0.03–64	32–>64	0 (0)	0 (0)	197 (100)
Gentamicin	0.125–32	4–>32	7 (3.55)	16 (8.13)	174 (88.32)
Amikacin	0.25–128	4–>128	12 (6.09)	31 (15.74)	154 (78.17)
Minocycline	0.125–32	0.25–2	197 (100)	0 (0)	0 (0)
Doxycycline	0.25–32	0.5–16	189 (95.94)	7 (3.56)	1 (0.5)
Tetracycline	0.25–32	1–>32	5 (2.54)	14 (7.1)	178 (90.36)
Tigecycline	0.015–32	0.5–16	70 (35.53)	97 (49.24)	30 (15.23)
Ciprofloxacin	0.004–8	0.125–>8	95 (48.22)	49 (24.88)	53 (26.9)
Levofloxacin	0.008–16	0.125–>16	150 (76.14)	8 (4.06)	39 (19.8)
Trimethoprim-sulfamethoxazole	0.25–8+4.75–152	4/38–>8/152	2 (1.02)	0 (0)	195 (98.98)
Rifampin	0.25–32	0.25–32	185 (93.91)	3 (1.52)	9 (4.57)
Vancomycin	0.25–64	4–>64	1 (0.51)	146 (74.11)	50 (25.38)
Chloramphenicol	1–64	8–>64	11 (5.58)	73 (37.06)	113 (57.36)

R, resistant; S, susceptible; I, intermediate resistant.

Furthermore, on the basis of the acquired antibiotic resistance pattern, 177 (89.9%) isolates were resistant to at least nine antibiotics, and 195 (99%) isolates were resistant to at least seven antibiotics. All isolates were resistant to β-lactams, including piperacillin, piperacillin-tazobactam, ceftazidime, cefepime, meropenem, and imipenem. High resistance rates were observed for piperacillin (98; 49.8%) in contrast to the decreased resistance when in combination β-lactamase inhibitors, namely, piperacillin-tazobactam (57; 28.9%). Only one and two strains were susceptible to ceftazidime and cefepime, respectively. Moreover, 196 (99.5%) isolates were resistant to imipenem, and 195 (99.5%) isolates were resistant to meropenem, which may be tricky for clinical treatment. *Elizabethkingia* isolates were extremely highly resistant to aztreonam; it was observed that none favored *in vitro* activity. They also exhibited high resistance rates to trimethoprim-sulfamethoxazole (195; 99%), tetracycline (178; 90.4%), gentamicin (174; 88.3%), amikacin (154; 78.7%), and chloramphenicol (113; 57.3%), respectively.

Interestingly, consistently with previous studies, doxycycline, minocycline, and rifampin inhibited >90% of all *E. anophelis* isolates. In particular, minocycline was more active compared with doxycycline and tigecycline [susceptible rates, 197 (100%) versus 189 (95.9%) and 70 (35.5%), respectively]. In this study, favored *in vitro* activity of fluoroquinolones was also observed, and the susceptibility rate for levofloxacin was higher than that of ciprofloxacin. In contrast, a significant difference was noted between the susceptibility rates of *E. anophelis* against levofloxacin (150; 76.1%) and ciprofloxacin (95; 48.2%), respectively. Taken together, these results evidently suggested that the bacteria were dangerous and highly resistant to the antibiotics. Although *E. anophelis* was resistant to a series of antibiotics, minocycline, doxycycline, and rifampin showed potent *in vitro* activity against this pathogen. Our findings provide potential alternative treatment options for *E. anophelis* infections.

### Biofilm formation of multidrug-resistant bacteria

Among the MDR behavior, each *E. anophelis* strain was screened for the ability to form biofilm. A simple culture plate assay was performed for the positive biofilm effect in tested *E. anophelis*. Compared with any other method, this assay is the most reliable and most straightforward method for identifying biofilm formation. In previous studies, this assay was found highly suitable for current research on the detection of biofilm formation in Gram-negative bacteria. *E. anophelis* strains collected in this study were highly susceptible to crystal violet observation. On the basis of the results of the culture plate, biofilm-positive *E. anophelis* were divided into three groups, namely, weak, moderate, and strong biofilm formers, and results are presented in **Figure S1**. In this study, our data revealed that all clinical *E. anophelis* isolates 197(100%) were biofilm-positive, with OD values >ODc at 595 nm. Moreover, 80 (40.6%) isolates tested positive for strong biofilm formation, with OD >4ODc at 595 nm, and four (Ea109, Ea124, Ea131, and Ea143) had the highest OD values, with values >1.5 (**Figure S1**). Of the 197 tested isolates, only 35 (17.8%) were weak biofilm formers, and 82 (41.6%) isolates were moderate biofilm formers. *E. anophelis* was strongly adherently after culture at 37°C in MHB.

Although the ability of *Elizabethkingia* to form biofilm was previously demonstrated, it must be pointed out that the percentage of biofilm-forming strains of *E. anophelis* was observed in the present investigation. We next compared the biofilm formation level (OD595) among the strains from different sources (**Figure S2**). There were significant differences among the other groups. **Figure S2** shows the percentage of strong, moderate, and weak biofilm formation levels in isolates from sputum, blood, abdominal fluid, CSF, and clinical/other strains. In contrast, sputum samples showed the highest (p < 0.05) percentage of strong biofilm-forming strains, whereas weak biofilms were formed mainly in bloodstream infection strains (p < 0.05). In addition, all strains isolated from abdominal and fluid CSF formed moderate biofilms. The different biofilm-forming ability for different origin isolates is still unclear, and further studies are needed to explain these findings.

**Figure 1:**
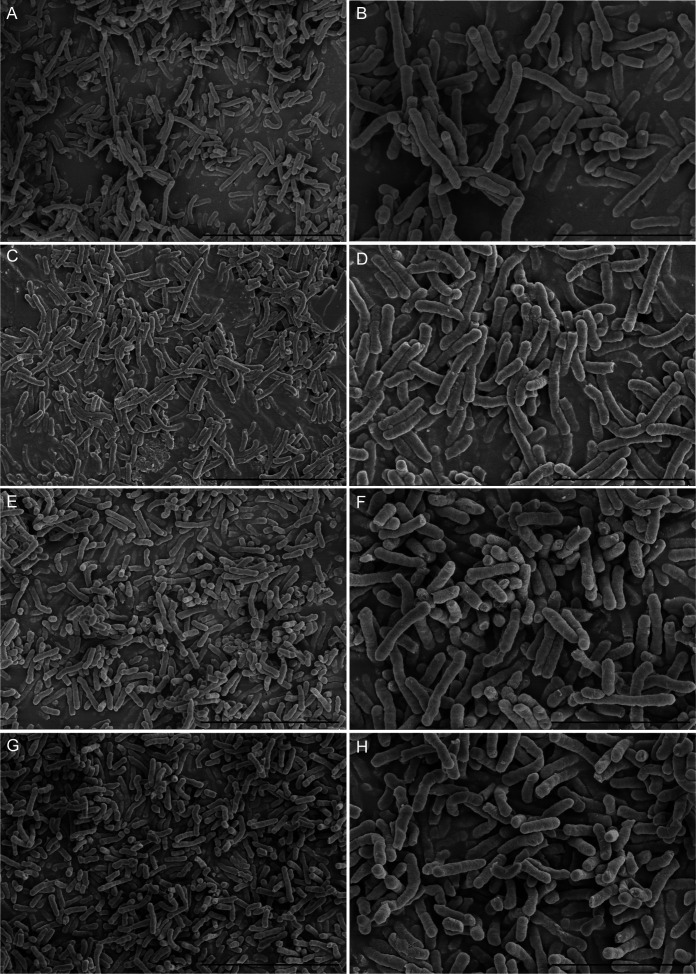
Scanning electron microscopy imaging analysis of four *E. anophelis* biofilm formations. The four strains were as follows: weak biofilm-forming strain SKL 051060 **(A, B)**; moderate biofilm-forming strain SKL014219 **(C, D)**; strong biofilm-forming strain SKL067015 **(E, F)**; and SKL068512 **(G, H)**. Among them, the left part of the figure **(A, C, E, G)** is the imaging result at 5,000-fold magnification (5.00k SE), bar = 10.0 µm; the right part of the figure **(B, D, F, H)** is the image result of 10,000-fold magnification (10.00k SE), bar = 5.00µm. The data show that the cell morphology is intact and densely stacked, and layered bacteria can be observed.

Furthermore, results of the tissue culture plate were cross-checked by the SEM analysis method. Eight *E. anophelis* sample clones were selected randomly for investigation by light microscopy, starting from the surface of the glass slide and scanning several planes interspersed by short distances to visualize biofilm architecture and microbial behavior throughout the depth of the individual flow chambers. **Figure 1** displays the *in vitro* biofilm formation results by four selected strains studied by SEM. Microcolonies merged to form a thick, complex biofilm structure across the entire surface of the coverslips. Each of these four strains showed thick biofilms, as densely stacked and layered bacteria were observed. In addition, differences in biofilm structure and cover channel surface were observed between stronger and weaker biofilm-forming strains. Compared with weaker biofilm-forming strains, the others contained thicker biofilms, and their bacteria were more densely stacked and layered (**Figure 1**).

### Correlation between resistance and biofilm formation capability

**Figure 2:**
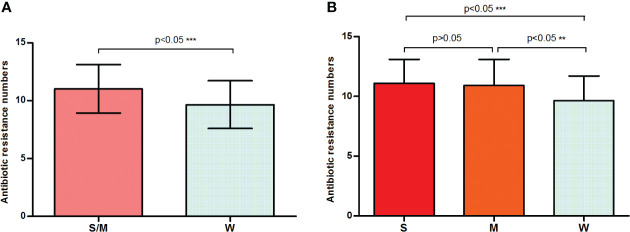
A bar graph displays the relationship between biofilm formation intensity and antimicrobial resistance. **(A)** Strong or moderate biofilm-forming strains presented a significantly higher average number of resistances than the weak producers. **(B)** The discrepancy in antimicrobial resistance among weak, moderate, and strong biofilm-producing isolates. W, weak biofilm formation; M, moderate biofilm formation; S, strong biofilm formation; S/W, strong or moderate biofilm formation. ** means significant at 0.01 level, *** means significant at 0.001 level.

One noteworthy point is that strong or moderate biofilm formers presented a statistically significantly higher (p = 0.0006) average number of resistances (11.01 ± 0.1643) compared with the weak formers (9.657 ± 0.3477) (**Figure 2A**). **Figure 2B** shows that the average number of resistances per strain between strong (11.10 ± 0.2247) and moderate (10.93 ± 0.2403) biofilm formers was not statistically significant (p > 0.05). Furthermore, both were statistically significant (p < 0.05) higher than that in weak biofilm formation classes (**Figure 2B**). This is the first time that a direct relationship has been reported between antibiotic resistance and biofilm formation in *E. anophelis*. Further studies are needed to support these findings.

**Figure 3:**
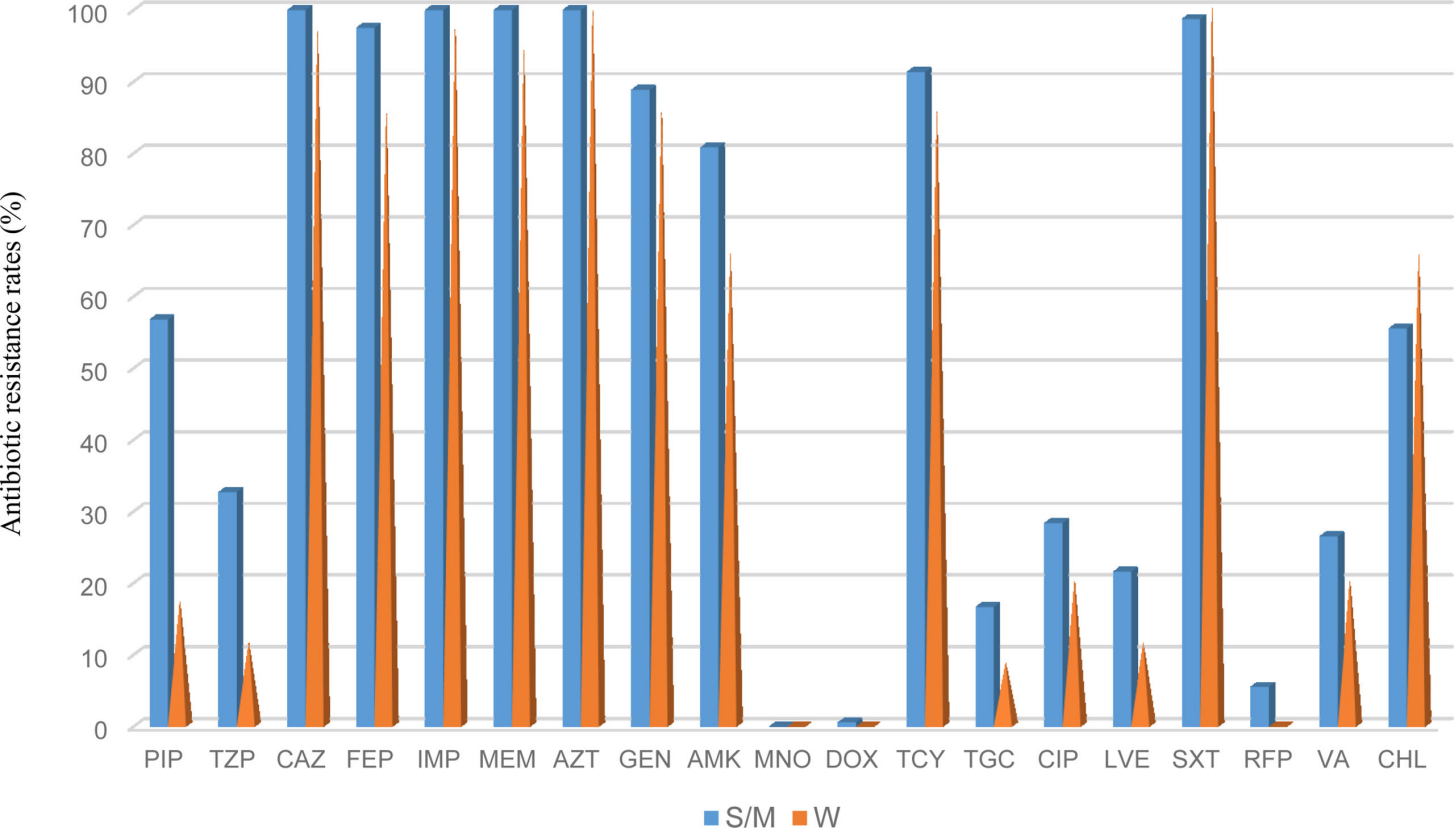
The frequency of antibacterial resistance in strong/moderate biofilm formation and weak biofilm producer *E. anophelis* isolates. S/W, strong or moderate biofilm formation; W, weak biofilm formation. PIP, piperacillin; TZP, piperacillin-tazobactam; CAZ, ceftazidime; FEP, cefepime; IPM, imipenem; MEM, meropenem; AZT, aztreonam; GEN, gentamicin; AMK, amikacin; MNO, minocycline; DOX, doxycycline; TCY, tetracycline: TGC, tigecycline; CIP, ciprofloxacin; LVX, levofloxacin; SXT, trimethoprim-sulfamethoxazole; RFP, rifampin; VA, vancomycin; and CHL, chloramphenicol.

**Figure 4:**
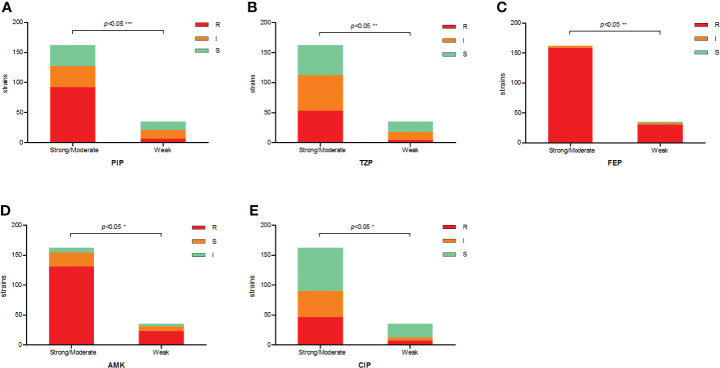
The correlation between antibiotic resistance and biofilm-forming capacity of clinical *E. anophelis* isolates to five antibiotics. **(A–E)** For piperacillin (PIP), piperacillin-tazobactam (TZP), cefepime (FEP), amikacin (AMK), and ciprofloxacin (CIP). Susceptible isolates tended to form weaker biofilms than non-susceptible isolates. Significant differences were detected between groups. The p-values obtained by Mann–Whitney analysis were as follows: piperacillin (p < 0.0001), piperacillin-tazobactam (p = 0.0088), cefepime (p = 0.0022), amikacin (p = 0.0442), and ciprofloxacin (p = 0.0461). * means significant at 0.05 alpha level, ** means significant at 0.01 level, *** means significant at 0.001 level.

The antimicrobial resistance pattern of *E. anophelis* isolates among strong, moderate, and weak biofilm formers is shown in **Table 2**. In general, the antimicrobial resistance rates among strong and moderate biofilm-forming *E. anophelis* strains were significantly higher than that of weak biofilm-forming isolates (**Table 2** and **Figure 3**). In particular, for ceftazidime, cefepime, imipenem, and meropenem, susceptible strains were only detected in bacteria that showed weak biofilm formation. Similarly, the isolates resistant to doxycycline or rifampin were merely found in strong and moderate biofilm-forming *E. anophelis*. A discrepancy was observed in the correlation between the degree of biofilm formation and antimicrobial resistance rates in most antibiotics from different classes, including piperacillin, piperacillin-tazobactam, ceftazidime, cefepime, meropenem, gentamicin, amikacin, doxycycline, tetracycline, tigecycline, ciprofloxacin, levofloxacin, rifampin, and vancomycin. However, the correlation could not be distinguished in case of aztreonam, minocycline, and trimethoprim-sulfamethoxazole (**Table 2** and **Figure 3**). Furthermore, after analyzing 12 antibiotics by the Kruskal–Wallis test, the difference between biofilm formation (among strong, moderate, and weak) and the proportion of antimicrobial-resistance was confirmed statistically significant (p < 0.05) in case of piperacillin, piperacillin-tazobactam, cefepime, amikacin, and ciprofloxacin, respectively (**Figure 4**).

**Table 2:** The relationship among biofilm-forming ability of *E. anophelis* with antibiotic resistance pattern.

Biofilm formation ability	Strong (n = 80)			Moderate (n = 82)			Weak (n = 35)		
Antibiotics	R No. (%)	I No. (%)	S No. (%)	R No. (%)	I No. (%)	S No. (%)	R No. (%)	I No. (%)	S No. (%)
Piperacillin	56 (70)	17 (21.25)	7 (8.75)	36 (43.9)	18 (21.95)	28 (34.15)	6 (17.14)	15 (42.86)	14 (40)
Piperacillin-tazobactam	29 (36.25)	38 (47.5)	13 (16.25)	24 (29.27)	21(25.61)	37(45.12)	4 (11.43)	14 (40)	17(48.57)
Ceftazidime	80 (100)	─	─	82 (100)	─	─	34 (97.14)	─	1 (2.86)
Cefepime	80 (100)	─	─	78 (95.12)	4 (4.88)	─	30 (85.71)	3 (8.57)	2 (5.72)
Imipenem	80 (100)	─	─	82 (100)	─	─	34 (97.14)	─	1(2.86)
Meropenem	80 (100)	─	─	82 (100)	─	─	33 (94.29)	─	2 (5.71)
Aztreonam	80 (100)	─	─	82 (100)	─	─	35 (100)	─	─
Gentamicin	74 (92.5)	5 (6.25)	1 (1.25)	70 (85.37)	8 (9.75)	4 (4.88)	30 (85.72)	3 (8.57)	2 (5.71)
Amikacin	67 (83.75)	10 (12.5)	3 (3.75)	64 (78.05)	13 (15.85)	5 (6.1)	23 (65.71)	8 (22.86)	4 (11.43)
Minocycline	─	─	80(100)	─	─	82(100)	─	─	35(100)
Doxycycline	─	2 (2.5)	78 (97.5)	1 (1.22)	4 (4.88)	77 (93.9)	─	1 (2.86)	34 (97.14)
Tetracycline	69 (86.25)	8 (10)	3 (3.75)	79 (96.34)	3 (3.66)	─	30 (85.71)	3 (8.57)	2 (5.72)
Tigecycline	10 (12.5)	44 (55)	26 (32.5)	17 (20.73)	37 (45.12)	28 (34.15)	3 (8.58)	16 (45.71)	16 (45.71)
Ciprofloxacin	18 (22.5)	25 (31.25)	37 (46.25)	28 (34.15)	19 (23.17)	35 (42.68)	7 (20)	5 (14.29)	23 (65.71)
Levofloxacin	14 (17.5)	─	66 (82.5)	21(25.61)	6 (7.32)	55 (67.07)	4 (11.43)	2 (5.71)	29 (82.86)
Trimethoprim- sulfamethoxazole	80 (100)	─	─	80(97.56)	─	2 (2.44)	35(100)	─	─
Rifampin	3 (3.75)	1 (1.25)	76 (95)	6 (7.32)	1(1.22)	75 (91.46)	─	1(2.86)	34 (97.14)
Vancomycin	31 (38.75)	48 (60)	1 (1.25)	12 (14.63)	70 (85.37)	─	7 (20)	28 (80)	─
Chloramphenicol	37 (46.25)	37 (46.25)	6 (7.5)	53 (64.63)	27 (32.93)	2 (2.44)	23 (65.71)	9 (25.72)	3 (8.57)

R, resistant; S, susceptible; I, intermediate resistant.

## Discussion

*E. anophelis* is an emerging pathogen that will cause life-threatening nosocomial infections in humans with compromised immune systems. Recently, *E. anophelis* infections in humans are increasing in many countries and showed high mortality, which consolidated the importance of early identification and treatment (Janda and Lopez, 2017; Lin et al., 2019). Furthermore, many global *E. anophelis* outbreak infections have been uncovered in recent years (Teo et al., 2013; Navon et al., 2016; Perrin et al., 2017; Lin et al., 2018a; Choi et al., 2019). Therefore, further exploration of antimicrobial resistance and virulence mechanisms in *E. anophelis* are of utmost importance. In the present study, the antimicrobial susceptibility characteristics and biofilm formation of nosocomial *E. anophelis* isolates were investigated, which were confirmed and obtained in China.

First, we found that *E. anophelis* isolates exhibited high MDR. The strains used in this study showed stabilized degrees of MDR to the tested antibiotics. Corresponding to previous reports, they were resistant to many commonly used antibacterial drugs, including penicillin, cephalosporin, carbapenem, aminoglycoside, fluoroquinolone, tetracyclines, sulfonamide, and carbapenem (Chen et al., 2015; Han et al., 2017; Chew et al., 2018; Lin et al., 2018a; Lin et al., 2018b; Choi et al., 2019). In this study, the antimicrobial resistance rates observed were as follows: piperacillin (49.8%), piperacillin-tazobactam (93%), ceftazidime (99.5%), imipenem (99.5%), meropenem (99%), aztreonam (100%), gentamicin (88.3%), amikacin (78.2%), tetracycline (90.4%), tigecycline (15.2%), ciprofloxacin (26.9%), levofloxacin (19.8%), trimethoprim-sulfamethoxazole (98.9%), vancomycin (25.4%), and chloramphenicol (57.4%), respectively. As presented in previous reports, minocycline, doxycycline, rifampin, and levofloxacin were active against *E. anophelis in vitro* in our study (Wang et al., 2020; Chiu et al., 2021; Kuo et al., 2021), which may be the first choice of empirical medication for the clinical treatment of this bacterial infection. Interestingly, as reported in Taiwan and by others, tigecycline, the derivative of minocycline, showed inferior antimicrobial activity in the present study (Jian et al., 2019; Chang et al., 2021; Kuo et al., 2021).

However, inconsistent AST results were reported for some antibiotics, especially for piperacillin-tazobactam, levofloxacin, rifampin, and vancomycin. For an *Elizabethkingia* infection, successful treatment has been described using piperacillin-tazobactam. An infant’s unusual presentation of *E. anophelis* infection indicated the pathogen was sensitive only to piperacillin-tazobactam (Mantoo et al., 2021). After antibiotic treatment was changed to piperacillin-tazobactam, the patient soon recovered and was discharged (Mantoo et al., 2021). According to a report by Wang et al., the antimicrobial susceptibilities of piperacillin-tazobactam were high at 86.5% (Wang et al., 2020). Moreover, in another report, it was also demonstrated that the combinations showed reasonable *in vitro* activity with a 71.8% susceptibility rate (Chang et al., 2021). In contrast, a significant difference was observed in our study. Only 34.01% of agent was active against *E. anophelis* isolates *in vitro*.

In line with the data in the present study, Chiu et al. revealed that piperacillin-tazobactam was notoriously active against 84 *E. anophelis* isolates *in vitro*. No susceptibility strain was found (Chiu et al., 2021). The variability in fluoroquinolone susceptibility has also been detected in the majority literature. Against levofloxacin and ciprofloxacin, different previous reports showed a different susceptibility of the *E. anophelis* pathogen. Although favored high *in vitro* activity of levofloxacin was observed in our study, the susceptibility rate of ciprofloxacin was much lower than that. In agreement with our results, in previous studies, it was concluded that, compared with levofloxacin, ciprofloxacin exhibited inferior activity against *E. anophelis* (Burnard et al., 2020; Tang et al., 2021). In other reports, when comparing our results, a significant difference was observed between the susceptibility rates of *E. anophelis* against ciprofloxacin (92% and 100%, respectively) (Lau et al., 2016; Perrin et al., 2017). In contrast, both ciprofloxacin and levofloxacin exhibited poor activity against *E. anophelis* isolates in hospitals in South Korea and Taiwan (Han et al., 2017; Lin et al., 2018a). These two AST results indicated that all the susceptibility rates were less than 30%, which is contradictory to our and previous findings (Han et al., 2017; Lin et al., 2018a).

It has been reported that the rifampin agent is less active against Gram-negative bacilli due to its weaker ability to readily penetrate the outer membrane of these pathogens (Goldstein, 2014). Chang et al. found that rifampin is unproductive to against *E. anophelis*, and the susceptibility rate was only 20.5% (Chang et al., 2021). Nevertheless, in most other studies, it was reported that rifampin is potent in fighting *E. anophelis* bacteria; even more than 95% of strains remained sensitive (Han et al., 2017; Seong et al., 2020; Wang et al., 2020; Chiu et al., 2021). Corresponding to these reports, rifampin also showed high *in vitro* activity inhibiting *E. anophelis* in our study. For rifampin, the underlying mechanism of high effectiveness to confront *E. anophelis* is yet known and warrants further investigation. Vancomycin has been suggested as a potential therapy for *Elizabethkingia* infections, particular in meningitis (Han et al., 2017; Jean et al., 2017; Lin et al., 2019; Seong et al., 2020). Therefore, in our study, *E. anophelis* strains were screened against vancomycin, but only 0.51% of susceptible isolates were detected. In line with our results, vancomycin is ineffective in the treatment of *Elizabethkingia* infection and has also been observed in previous reports (Han et al., 2017; Wang et al., 2020; Chang et al., 2021; Chiu et al., 2021; Kuo et al., 2021). Unfortunately, these results suggest that the choice of the abovementioned empirical antimicrobial therapy for *E. anophelis* remains controversial, and further investigations are urgently needed to determine the optimal antibiotics for treating this bacterium infection.

In the present study, all detected *E. anophelis* isolates were capable of forming biofilms. As mentioned, 40.6%, 41.6%, and 31% of clinical isolates formed strong, moderate or weak biofilms, respectively. These findings were comparable with the results reported by Tang et al., who noted that biofilm formation was high at 96.7%, as only one *E. anophelis* isolate tested negative for biofilm formation (Tang et al., 2021). Moreover, it was indicated that more than a quarter of the isolates tested positive for strong biofilm formation, with the highest OD values reaching 2.0 (Tang et al., 2021). In contrast, the biofilm-forming ability of the strain that we isolated was superior. Next, our data showed that the biofilm formation was higher in sputum samples, whereas weak biofilms were mainly formed in bloodstream infection *E. anophelis* strains. However, there may be a limitation because no comparable study can be obtained of *E. anophelis*. Thus, future investigations among *E. anophelis* strains from different sources are warranted.

In previous studies, it was documented that biofilm-forming bacteria could reduce antibiotic susceptibilities and be more resistant to the antibacterial agent than non–biofilm-forming strains (Yang et al., 2019; Shenkutie et al., 2020; Hashemzadeh et al., 2021). By comparing the results in this study, a similar outcome was found for the *E. anophelis* pathogen. The strains that obtained strong or moderate biofilm formation presented statistically significant higher resistances compared with the weak producers (p < 0.05), especially for piperacillin, piperacillin-tazobactam cefepime, amikacin, and ciprofloxacin. In a previous study, it was demonstrated that biofilm formation in MDR *Staphylococcus saprophyticus* isolates was significantly higher than that of non MDR *Staphylococcus saprophyticus* isolates. At the same time, no significant relationship was detected between MDR and biofilm formation intensity (strong, moderate, and weak) (Hashemzadeh et al., 2021). The result was similar to our findings, and in the present study, no significant relationship was observed between MDR, XDR, and biofilm-forming intensity. Compared with previous investigations, our results indicated that the correlation between the antibacterial agent and biofilm strength was different among the different antibiotics (Fauzia et al., 2020; Shadkam et al., 2021). For gentamicin, tetracycline, vancomycin, and chloramphenicol, no significant difference in biofilm formation between sensitive and resistant isolates was observed. We presume that this was partially due to the difference sample size, which may affect the statistical analysis. For example, 174 isolates were non-susceptible to gentamicin, and only seven were susceptible. For tetracycline, 178 isolates were non-susceptible, and only five were susceptible. It is important to understand and clarify the biofilm formation and antibiotic resistance mechanism of *E. anophelis* and identify effective method of blocking-up biofilm for the prevention and treatment of this species. However, previous studies in other bacteria have documented that biofilm-acquired drug resistance is complicated and likely involves the expression of virulence genes and efflux pumps, growth and metabolic adaptations, horizontal gene transfer, gene mutation, stress response, and others factors (Bonifait et al., 2008; Soto, 2013; Dutkiewicz et al., 2018; Yi et al., 2020). Thus, outcomes from our investigation should be interpreted with caution, because the methods utilized in this investigation cannot be used to adequately assess biofilm-mediated MDR mechanisms.

To overcome this limitation, more thorough investigations to study the relationship between biofilm formation and antibacterial resistance, including faster conjugative plasmid transfer or multiplication of specific regulatory horizontal gene transfer genes, should be conducted in future studies to clarify these underlying mechanisms of action. In conclusion, studies on *E. anophelis* biofilms are still in its infancy. The result obtained in this study may be an essential stepping-stone for considering biofilm formation in drug susceptibility testing to improve the antimicrobial therapy effect with *E. anophelis* infections.

## Data availability statement

The original contributions presented in the study are included in the article/**Supplementary Material**. Further inquiries can be directed to the corresponding authors.

## Ethics Statement

No potentially identifiable human images or data was presented in this study and the surveillance was part of the hospital infection control.

## Author contributions

Conceptualization: SH and BZ; data curation: SH, YL, and HX; formal analysis: SH and HX; funding acquisition: BZ and YX; methodology: BZ and YX; project administration: SH and YL; resources: BZ and YX; software: HX; supervision: BZ; writing—original draft preparation: SH; writing—review and editing: BZ and YX. All authors have read and agreed to the published version of the manuscript.

## Funding

This work was supported by the Zhejiang Provincial Natural Science Foundation of China (Grant No. LQ21H190002), the Zhejiang Provincial Natural Science Foundation of China (Grant No. LQ20H080002), and the National Natural Science Foundation of China (No. 82072314). The Research Project of Jinan Microecological Biomedicine Shandong Laboratory (JNL-2022006B and JNL-2022011B), the Fundamental Research Funds for the Central Universities (2022ZFJH003), and CAMS Innovation Fund for Medical Sciences (2019-I2M-5-045).

## Acknowledgments

SEM imaging was performed in the State Key Laboratory of Rice Biology of Zhejiang University, with the support of Dr. Fang Wang.

## Conflict of interest

The authors declare that the research was conducted in the absence of any commercial or financial relationships that could be construed as a potential conflict of interest.

## Publisher’s note

All claims expressed in this article are solely those of the authors and do not necessarily represent those of their affiliated organizations, or those of the publisher, the editors and the reviewers. Any product that may be evaluated in this article, or claim that may be made by its manufacturer, is not guaranteed or endorsed by the publisher.
