# Supplementary material for: Biofilm formation and antibiotic sensitivity in Elizabethkingia anophelis
Source: Front Cell Infect Microbiol. 2022 Jul 28;12:953780. doi: 10.3389/fcimb.2022.953780 (PMC9366890; doi:10.3389/fcimb.2022.953780)
Supplement: Supplementary file 2 [file DataSheet_1.pdf]

Supplementary Material

1. Supplementary Figure1

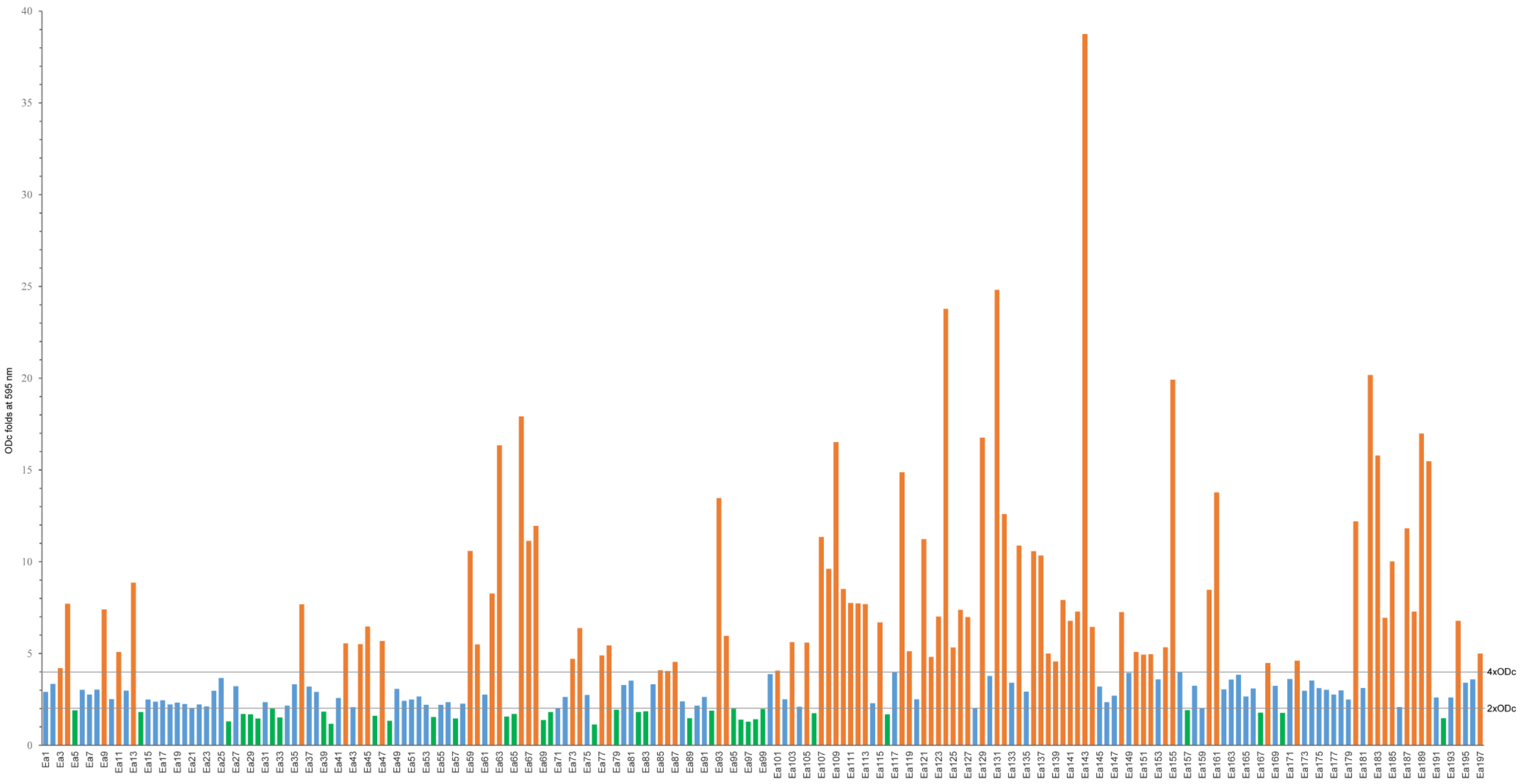

Figure S1. Distribution of biofilm formation in 197clinical *E. anophelis*. The X-axis represents the *E. anophelis* bacteria and the Y-axis means the optical density by using the crystal violet assay to screen biofilm formation among the isolates. The differentiated criteria are as the following, OD<ODc: non-biofilm formation; OD >ODc–2xODc: weak biofilm formation; OD >2xODc–4xODc: moderate biofilm formation; OD >4xODc: strong biofilm formation.

2. Supplementary Figure2

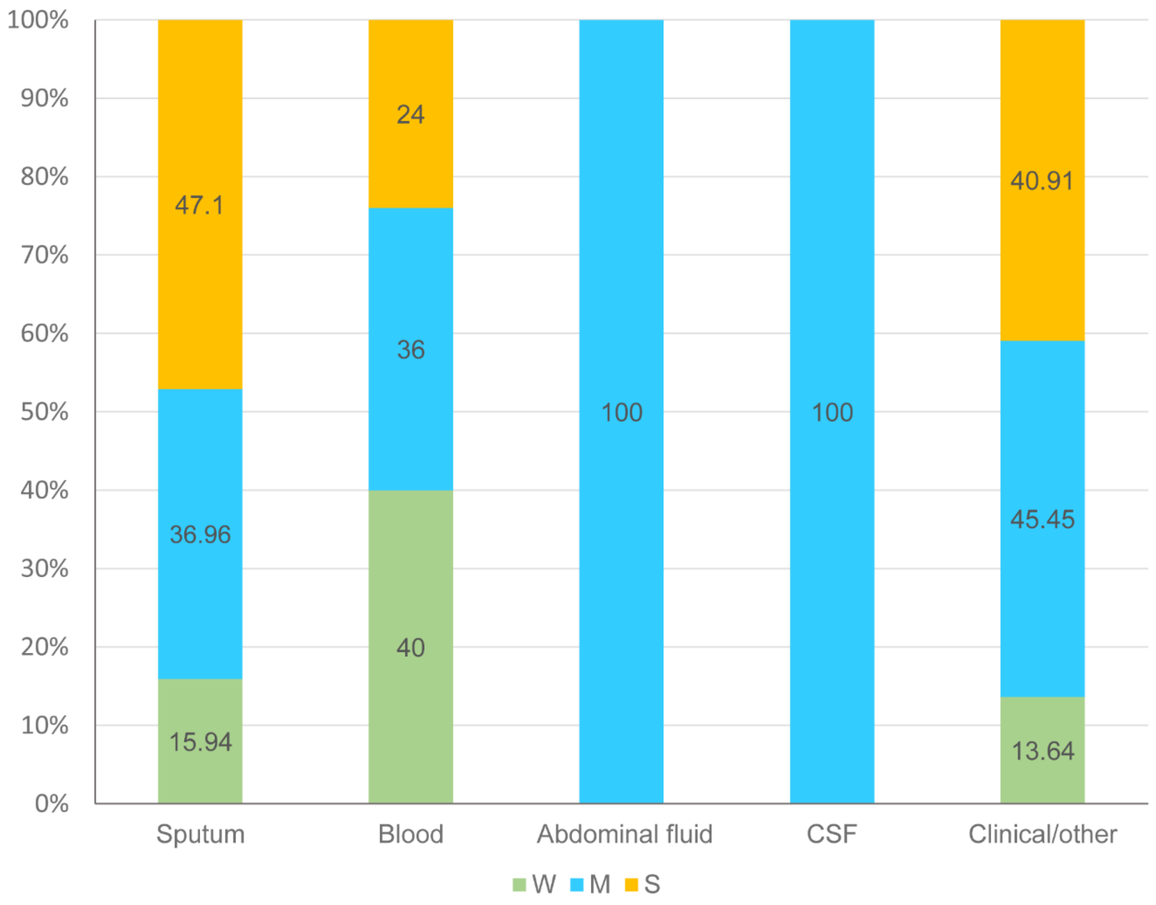

Figure S2. Percentages of strong, moderate and weak biofilm-forming *E. anophelis* were examined in sputum, blood, abdominal fluid, CSF and clinical/other samples. W: weak biofilm formation; M: moderate biofilm formation; S: strong biofilm formation.
